# Detecting Risk of Low Health Literacy in Disadvantaged Populations Using Area-based Measures

**DOI:** 10.5334/egems.191

**Published:** 2017-12-15

**Authors:** Andrew J. Knighton, Kimberly D. Brunisholz, Samuel T. Savitz

**Affiliations:** 1 Intermountain Healthcare, US; 2 University of North Carolina at Chapel Hill, US

**Keywords:** health literacy, deprivation, poverty, learning healthcare system, health services research

## Abstract

**Introduction:**

Socio-economic status (SES) and low health literacy (LHL) are closely correlated. Both are directly associated with clinical and behavioral risk factors and healthcare outcomes. Learning healthcare systems are introducing small-area measures to address the challenges associated with maintaining patient-reported measures of SES and LHL. This study’s purpose was to measure the association between two available census block measures associated with SES and LHL. Understanding the relationship can guide the identification of a multi-purpose area based measure for delivery system use.

**Methods:**

A retrospective observational design was deployed using all US Census block groups in Utah. The principal dependent variable was a nationally-standardized health literacy score (HLS). The primary explanatory variable was a state-standardized area deprivation index (ADI). Statistical methods included linear regression and tests of association. Receiver operating characteristic (ROC) analysis was used to develop LHL criteria using ADI.

**Results:**

A significant negative association between the HLS and the ADI score remained after adjusting for area-level risk factors (β: –0.21 (95% CI: –0.22, –0.19) p < .001). Eighteen block groups (<1%) were identified as having LHL using HLS. A combination of three or more ADI components correlated with LHL predicted 78% of HLS LHL block groups and 35 additional block groups not identified using HLS (c-statistic: 0.64; 95% CI: 0.62, 0.66).

**Conclusions:**

HLS and ADI use differing measurement criteria but are closely correlated. A state-based ADI detected additional neighborhoods with risk of LHL compared to use of a national HLS. An ADI represents a multi-purpose area measure of social determinants useful for learning health systems tailoring care.

## Introduction

Due to higher prevalence estimates of risk factors, socially disadvantaged persons have a considerable need for health-related information. In 2004, the Institute of Medicine suggested that social risk factors, including socio-economic position, residential and community context, have a potential causal relationship with health literacy [1]. Health literacy is defined as the “degree to which individuals have the capacity to obtain, process, and understand basic health information and services needed to make appropriate health decisions” and is essential to navigating the health care system [2,3]. Additionally, socio-economic factors along with health literacy are both directly associated with clinical and behavioral risk factors, health care use and health care outcomes [4].

Low health literacy is not defined by patient education level or language barriers alone. Health system complexity, medical jargon and exposure to novel health concepts have the potential to exceed a patient’s literary skills, even among literate adults [5]. Low health literacy, however, represents a particular challenge in disadvantaged populations, many of whom may possess multiple risk factors for low health literacy. Low health literacy is associated with poor understanding of the origin, nature and course of health problems including lower screening, lower adherence rates and inability to navigate the health system in these populations [4]. Health literacy represents a barrier to reducing gaps in the quality of care and improving health outcomes in disadvantaged populations [4].

Measuring health literacy through validated instruments, such as the REALM-SF [6] or the S-TOFHLA [7] is challenging for clinicians to administer. As such, health care systems typically do not assess patients’ health literacy, which leads to poor identification and difficulty tailoring interventions at the point-of-care. Learning health care systems are introducing area-based measures to identify patients from disadvantaged neighborhoods with underlying social risk factors that may signal increased risk of low health literacy [8]. The potential benefits of using an area-based measure over patient-reported information include lower cost, more consistent source data capture and improved comparability of information over time. While prior research demonstrates an association between individual patient disadvantage and low health literacy, no studies have examined this relationship at a neighborhood or area level.

The primary objective of this study was to measure the correlation between an area-based measure of disadvantage using an area deprivation index (ADI) or one of its components and a neighborhood-level measure of health literacy. Given the findings, criteria for detecting disadvantaged neighborhoods at risk for low health literacy was developed using components of an ADI. Differentiating risk factors for health literacy allows learning health care systems to better adapt interventions to address the impact of health literacy on outcomes. Understanding the relationships that exist between area-based measures can also guide the potential identification of a multi-purpose area based measure for delivery system use.

## Methods

We utilized a retrospective, observational study design to determine the association of neighborhood deprivation with levels of health literacy among Census Block Groups (CBGs) in the state of Utah.

### Study Population/Study Setting

CBGs identified in the state of Utah were the unit of analysis for this study. Census data, including ADI calculations, were drawn from the Intermountain Healthcare Enterprise Data Warehouse based upon 2013 American Community Survey data published by the U.S. Census Bureau. CBGs were excluded for study if there were no residents found within the selected geographical area. This study was limited to publically reported data for analysis and thus, approval from Intermountain Healthcare’s institutional review board was not required.

### Study Measurement

**Principal Measures.** The principal dependent variable measured in this study was the health literacy score (HLS) by U.S. Census block group. The HLS was developed by researchers at University of North Carolina [9,10] using a predictive model based on the 2003 National Assessment of Adult Literacy (NAAL) [11]. The NAAL was an in-person assessment of English language literacy among a nationally representative sample of U.S. adults age 18 and over (n = 17,466). HLS groupings include four ordinal categories (i.e., below basic, basic, intermediate, and proficient). As described in Appendix 1, HLS intervals are associated with the existence of the necessary health literacy skills to conduct certain health tasks. Mean health literacy scores by CBG were calculated based upon the predictive factors listed in Table 1. Demographic data to develop the HLS by block group was drawn from the 2010 US census and the 2011 5-year American Community Surveys (ACS) summary files. In addition to the ordinal categories stated above, HLS values were also grouped for this study by state-specific quintiles in order to improve its representativeness to those living within the state of Utah.

**Table 1:** Comparison of risk factors included in the area deprivation index and the health literacy score.

Singh area deprivation index components	Health literacy score

Median family income, $	Poverty status (income)
Income disparity	Race
Families below poverty level, %	Ethnicity
% population below 150% poverty threshold, %	Education
Single parent households with dependents <18, %	Sex
Households without a motor vehicle, %	Marital status
Households without a telephone, %	Age
Occupied housing units without complete plumbing, %	Metropolitan Statistical Area (MSA)
Owner occupied housing units, %	Language
Households with >1 person per room, %	Years in country
Median monthly mortgage, $	
Median gross rent, $	
Median home value, $	
Employed person 16+ in white collar occupation, %	
Civilian labor force unemployed (aged 16+), %	
Population aged 25+ with <9-year education, %	
Population aged 25+ with at least high school education, %	

The principal explanatory variable identified was relative area deprivation status, measured using an area deprivation index (ADI). The ADI is a geographic area-based measure of the disadvantaged position of residents relative to the society [12]. The ADI was calculated for the state of Utah using a measure developed by Singh [13] based upon 17 U.S. Census measures associated with mortality, including living conditions, income, unemployment and education listed in Table 1. Census measures were based upon the 2013 American Community Survey published by the U.S. Census Bureau. Because previous research suggests that neighborhood effects are non-linear, ADI scores were grouped by quintile with the lowest quintile associated with lower deprivation and higher socio-economic status [14,15]. Other anticipated risk factors for both area deprivation and lower health literacy were summarized by area-based measure included median age, sex (percent male), race (percent non-white), ethnicity (percent Hispanic) and residence (percent urban).

### Statistical Methods and Testing

Testing was conducted using multiple statistical tests. Univariate and multivariate ordinary least squares (OLS) regression with robust standard errors was used to analyze the association between HLS and ADI scores by CBG. Pearson’s chi-square (χ^2^) was then used to compare the relative grouping of CBGs by ADI quintile with HLS using Utah-specific HLS quintiles. Goodman and Kruskal’s gamma (γ) measured the strength of rank correlation between quintile groupings. To measure the association between individual ADI components and HLS, univariate OLS determined significance and Pearson’s correlation coefficient (ρ) measured the strength of association to identify components of ADI with the strongest associations with lower health literacy. Pearson’s chi-square (χ^2^) analysis was utilized to evaluate the association of the ADI’s most deprived 5 percent and 10 percent CBGs with each of the 17 individual measures with ‘basic’ HLS categorization as defined by NAAL. Goodman and Kruskall’s gamma (γ) determined the strength of the association. Finally, non-parametric Receiver Operating Characteristics (ROC) curves evaluated which individual ADI measure or combination of measures most closely predicted classification of ‘basic’ HLS and reported as c-statistics. For all analyses, a two-sided p-value ≤ .05 was considered statistically significant. All data were analyzed using Stata 12.0 (StataCorp LLC, College Station, Texas).

## Results

### Measuring the Study Population

In the state of Utah, 1690 CBGs were identified. Four CBGs did not have residents and were excluded from the final study results (n = 1686). Individual census measures were unavailable given CBG population size as prescribed by census data methodology [16]. As a result, actual CBG counts varied modestly when examining individual census measures.

### Utah Census Block Groups Characteristics

CBG characteristics for the state of Utah were included in Table 2 for comparison purposes. Reviewing the relative distribution of HLS for the state of Utah, 1668 block groups were identified as having ‘intermediate’ health literacy. Eighteen (18) block groups (~1.0 percent) were associated with ‘basic’ health literacy skills. None of the block groups received either a ‘below basic’ or ‘proficient’ score using the HLS ordinal interval rankings. In the state of Utah, the mean resident count per block group was 1,639 ± 864 (range: 25–11,672) individuals and age was 31.8 ± 7.7 years per CBG. Of Utah residences, 88.5 percent were urban, median family income was $68,793 ± $28,052, and 90.8 percent received a high school diploma. Overall, the distribution for CBG attributes were similar when comparing ADI quintiles and Utah-specific HLS quintiles. As expected, the lowest health literacy category had an older median age (32.2 ± 7.8 years vs. 30.2 ± 7.1 years), and a higher proportion of non-white (23 percent vs 19 percent; p < .001) and Hispanic (29 percent vs 24 percent; p < .001) populations when compared to the most deprived ADI quintile.

**Table 2:** U.S. census block group demographics comparing an area deprivation index (ADI) and health literacy score (HLS).

Block Attributes	Utah – Overall			Area Deprivation Index				Health Literacy Score						

	Count	Mean	SD	Quintile	Count	Mean	SD	Quintile	Mean	SD	Category	Count	Mean	SD

Age – Median	1686	31.8	7.73	Least	338	34.4	8.9	Most	28.9	8.3	Proficient	0		
				2	337	32.6	7.7	4	32.3	7.3	Intermediate	1668	31.8	7.7
				3	337	31.0	7.0	3	32.4	6.9	Basic	18	28.4	4.9
				4	337	30.7	6.9	2	33.1	7.5	Below basic	0		
				Most	337	30.2	7.1	Least	32.2	7.8				

Sex – % Male	1686	0.503	0.05	Least	338	50%	0.05	Most	50%	0.05	Proficient	0		
				2	337	50%	0.05	4	51%	0.04	Intermediate	1668	0.50	0.05
				3	337	50%	0.05	3	50%	0.05	Basic	18	0.52	0.06
				4	337	51%	0.05	2	50%	0.05	Below basic	0		
				Most	337	50%	0.06	Least	51%	0.06				

Race – % Nonwhite	1686	0.114	0.12	Least	338	7%	0.06	Most	8%	0.07	Proficient	0		
				2	337	8%	0.08	4	8%	0.07	Intermediate	1668	0.11	0.11
				3	337	11%	0.10	3	9%	0.08	Basic	18	0.53	0.26
				4	337	13%	0.12	2	10%	0.08	Below basic	0		
				Most	337	19%	0.17	Least	23%	0.18				

Ethnicity – % Hispanic	1686	0.128	0.14	Least	338	5%	0.07	Most	6%	0.06	Proficient	0		
				2	337	8%	0.08	4	7%	0.07	Intermediate	1668	0.12	0.13
				3	337	12%	0.10	3	10%	0.09	Basic	18	0.48	0.28
				4	337	15%	0.13	2	12%	0.10	Below basic	0		
				Most	337	24%	0.18	Least	29%	0.18				

Residence – % Urban	1686	0.885	0.29	Least	338	91%	0.24	Most	96%	0.13	Proficient	0		
				2	337	91%	0.24	4	94%	0.2	Intermediate	1668	0.89	0.29
				3	337	88%	0.29	3	86%	0.31	Basic	18	0.77	0.42
				4	337	85%	0.34	2	79%	0.38	Below basic	0		
				Most	337	88%	0.32	Least	88%	0.32				

Median family income	1673	$68793	$28052	Least	336	$105,045	$30,776	Most	$ 89,776	$36,603	Proficient	0		
				2	336	$78,370	$12,806	4	$81,296	$25,887	Intermediate	1655	$69214	$27897
				3	334	$65,252	$11,880	3	$67,213	$19,399	Basic	18	$30091	$8810
				4	333	$53,439	$12,241	2	$57,716	$13,979	Below basic	0		
				Most	334	$41,539	$11,440	Least	$48,323	$15,303				

Education – % high school diploma	1673	0.908	0.09	Least	336	97%	0.04	Most	97%	0.03	Proficient	0		
				2	336	95%	0.05	4	96%	0.04	Intermediate	1655	0.91	0.09
				3	334	92%	0.06	3	93%	0.05	Basic	18	0.64	0.12
				4	333	88%	0.08	2	90%	0.06	Below basic	0		
				Most	334	82%	0.12	Least	79%	0.11				

### Association between Area Deprivation Index and Health Literacy Score

**Figure 1:**
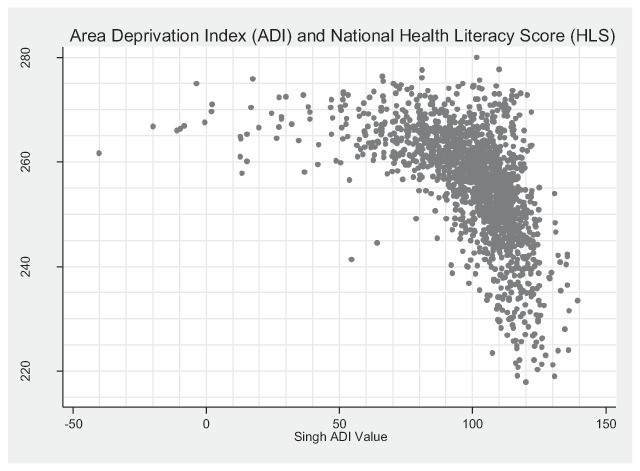
Association between area deprivation index and health literacy score by U.S. Census block group.

Figure 1 highlights the observed relationship between ADI and HLS by CBG. A multivariate regression analysis including potential confounding factors (percent non-white, percent Hispanic, percent urban and a quadratic term for median age), demonstrated a significant negative association between the HLS and the ADI score (β: –0.21; 95 percent CI: –0.22 to –0.19; p < .001). Excluding the ADI score, the initial model explained 57 percent of the variation in HLS (r-squared: 0.57). Addition of ADI in the final model significantly contributed to an 11 percentage points increase in the explained variation (r-squared: 0.68) (Likelihood Ratio test; p < .001).

**Figure 2:**
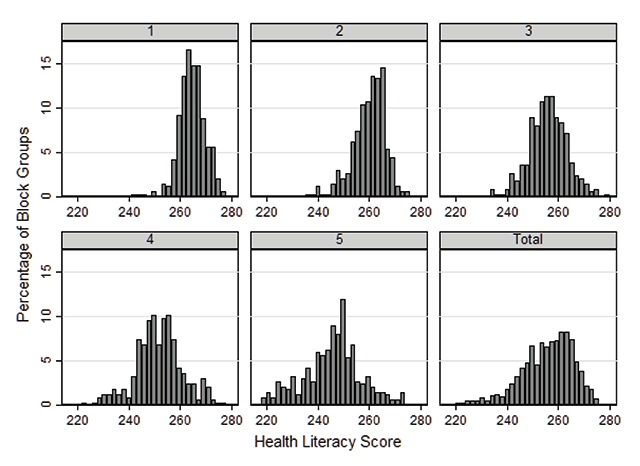
Percent distribution of health literacy score by U.S. Census block group by Singh area deprivation index quintile (least deprived = 1) and in total.

Figure 2 examines the distribution of HLS by ADI quintile and demonstrates increased variation in HLS as neighborhood deprivation increases. Additionally, 96 percent (n = 17/18) of CBGs in the HLS ‘basic’ category were also identified in the fifth or most deprived ADI quintile. A significant association was noted (p < .001) when comparing the relative ordinal groupings of ADI and HLS quintiles. Rank correlation noted a moderate to strong relationship between the two ordinal measures (γ = 0.65; asymptotic standard error = .017). Variation between ordinal rankings using the two methods was highest in the middle quintiles.

### Examining Individual Area Deprivation Index Components

All but one of the individual components of ADI (median rent per CBG) was significantly associated with the HLS (Table 3). As expected, stronger positive correlations (ρ > .50) were noted for CBGs with higher median family income and white-collar employment. Stronger negative correlations (ρ < –.50) were noted for CBGs with lower levels of high-school educated individuals as well as for CBGs with high levels of single-parent households and high levels of residents with less than 9^th^ grade education.

**Table 3:** Correlation and strength of association between component U.S. Census measures included in the Singh Area Deprivation Index (ADI) and an area-based health literacy score (continuous and categorical comparisons).

	Comparison of continuous measures	Comparison of categorical measures: bottom 5% ADI component measure (0/1) and basic literacy category (0/1)	

Area deprivation index (ADI) component measure	Health literacy score (ρ)	Basic literacy category (γ)	C-statistic (95% CI)

**Median family income, $**	0.54	.93	0.56 (95% CI: 0.53–0.58)
**Income disparity**	–0.37		
**Families below poverty level, %**	–0.41	.94	0.56 (95% CI: 0.54–0.59)
**% population below 150% poverty threshold, %**	–0.43		
**Single parent households with dependents <18, %**	–0.65		
**Households without a motor vehicle, %**	–0.34		
**Households without a telephone, %**	–0.22		
**Occupied housing units without complete plumbing, %**	–0.09		
**Owner occupied housing units, %**	0.23		
**Households with >1 person per room, %**	–0.39	.96	0.58 (95% CI: 0.55–0.60)
**Median monthly mortgage, $**	0.43		
**Median gross rent, $**	0.02^†^		
**Median home value, $**	0.54		
**Employed person 16 + in white collar occupation, %**	0.67		
**Civilian labor force unemployed (aged 16+), %**	–0.35		
**Population aged 25+ with <9-year education, %**	–0.61	.97	0.58 (95% CI: 0.55–0.60)
**Population aged 25+ with at least high school education, %**	–0.61	.95	0.58 (95% CI: 0.54–0.59)

ρ = Pearson correlation coefficient; γ = Goodman and Kruskall’s gamma. All ADI measures significantly correlate with health literacy score except as noted with ^†^ (p < .05). Only ADI measures with a γ > .95 in the bottom 10% were evaluated in the bottom 5%.

Looking specifically at the 18 block groups categorized by using NAAL as ‘basic’ health literacy, strength of correlation with those block groups in the upper or lower 5^th^ percentile was most prominent for education, income and housing categories (Table 3). Examination of each ADI component individually using a measure in the upper/lower 5^th^ percentile found little variation in the c-statistic across individual components in predicting NAAL ‘basic’ classification (Table 3). However, when identifying block groups based upon the presence of three or more high-risk components in ADI associated with low health literacy as noted in Table 3, a significant improvement in results over the use of an individual characteristics was observed (c-statistic: 0.64; 95 percent CI: 0.62–0.66). This combined approach would then detect 14/18 block groups initially identified as ‘basic,’ along with another 35 additional block groups that share similar area-level risk factors linked to low health literacy (representing about 3 percent of Utah CBGs).

## Discussion

This study contributes a novel quantitative comparison of two area-based measures while providing utility to health care systems evaluating the effects of area deprivation and health literacy on health care outcomes. Results show that the HLS and the ADI have a significant inverse correlation—lower HLS is associated with higher ADI. The results demonstrate that an area-based measure of deprivation was independently correlated with an area-based measure of health literacy after adjusting for area-level mean demographics including age, race, ethnicity and urban status. While these results are not unexpected, the reasoning for this association may be explained due to commonality of underlying risk factors and similarity in methodology for calculating both measures. Yet, area-level correlation between neighborhood disadvantage and neighborhood health literacy has not been confirmed in previous studies. Additionally, variation in HLS was greater at higher levels of deprivation. Taken together, these findings suggest that the HLS and ADI have similar relative values for block groups, but more divergence when deprivation is high.

As noted earlier, health literacy is dependent on both individual and systemic factors. Not all block groups with high deprivation in this study were linked to ‘basic’ health literacy. Low health literacy may be a function of several risk factors including communication skills, knowledge of health topics, culture, demands on the system, and an individual’s situation or context [17]. In this study, we demonstrated that ADI in conjunction with HLS may detect low health literacy that results from social disadvantage. By differentiating between risk factors for health literacy, a learning health care system can better adapt interventions to address the impact of literacy on outcomes. Further study is needed to evaluate how this proposed approach would work in care delivery.

These findings have implications for how delivery systems can target patients for interventions. Increasingly, delivery systems are attempting to address social determinants of health in the population they serve. Neighborhood deprivation has been shown to have a strong association with poor health outcomes [18]. This association can be partially explained by similar risk factors for both neighborhood deprivation and low health literacy [13,14,15,16,17,18,19]. Our analysis provides evidence that area level disadvantage and health literacy are closely correlated. Like deprivation, health literacy has also been linked to worse health outcomes [4] and may explain why patients from more deprived areas have poorer health care outcomes. While delivery systems have a limited ability to mitigate the impact of high deprivation, identifying circumstances where health care disparities may result from low health literacy linked to social disadvantage, treatment interventions may be actionable. These interventions might include tailored education, disease management programs and medication reviews [20].

One barrier to the use of HLS alone in identifying neighborhoods with low health literacy is that the nationally-weighted measure was too selective in identifying at-risk populations in Utah. In this study, only 1 percent of block groups in Utah were identified using HLS as ‘basic’ health literacy (versus 15 percent nationally). Yet, our analysis demonstrated that a number of Utah CBGs sharing similar at-risk characteristics associated with the NAAL ‘basic’ classification, including lower education attainment and lower income levels, were not identified as at risk for low health literacy using the HLS classification. This percentage (1 percent) may be too small for using a nationally-weighted HLS to detect local small-area variation. Alternatively, the use of a measure with less stringent criteria may be considered—including the use of a state-level standardized ADI, individual components of the ADI, or state-specific HLS quintiles. These alternative measures may better identify segments of the Utah population, particularly those coming from the most deprived neighborhoods, most likely affected by low health literacy. Using a combination of three or more ADI characteristics associated with categorization as ‘basic,’ an additional 35 CBGs were identified that share similar risk factors linked to low health literacy that could be considered in identifying patients from “at risk” neighborhoods.

Several limitations were identified within this study. First, the analysis was restricted to the state of Utah. We made this restriction because we have a detailed understanding of Utah’s demographic characteristics. This knowledge was important for evaluating the area-based measures. In addition, we were interested in the value of these measures to inform care delivery in Utah. This limitation means the findings may not generalize to the rest of the country. As noted, Utah had a much smaller proportion of block groups identified as basic/below basic (1 percent) than the U.S. average (15 percent). This may be due in part to population demographics. Using the HLS (including NAAL categorizations) may be more helpful for targeting interventions in regions with different demographics and a higher proportion of block groups identified as basic/below basic.

Second, the alternative approaches to detecting low health literacy using an ADI are weaker in terms of their conceptualization. NAAL categories relate to specific skills depicted by individuals in that group (Appendix 1) [21]. The alternative approaches sacrifice this relationship for better performance in identifying high-risk areas. This tradeoff is reasonable in the context of delivery systems attempting to target high-risk patients. However, the NAAL categories would be more appropriate when conducting research on the effect of health literacy.

Third, neighborhood-level health literacy data may not mirror individual-level SES-health literacy relationships. Given the potential effects of the ecologic fallacy, the area-based measure serves as only a proxy for individual health literacy. Validation of the published HLS measure was outside the scope of this paper. In contrast, the ADI is often used as the measure of interest instead of as a proxy for individual income. For health literacy, instruments such as the REALM or S-TOFHLA [6,7] or validated screening questions [22] would be more accurate in identifying individual patients. However, most delivery systems do not have the resources to administer these assessments to a large proportion of their patient population. In such cases, area-based measures represent a reasonable basis for initial stratification of the patient population to inform the use of scarce resources in delivering quality care.

## Conclusion

This study confirms that low health literacy correlates with high deprivation at an area level. While NAAL categorization appeared restrictive in identifying Utah CBGs at high risk of low health literacy, utility of an alternative approach using components of ADI identified additional neighborhoods with high risk of low literacy resulting from social disadvantage. Area-based measures for social determinants of health may aid learning health care systems in reducing disparities for patients that result from low health literacy through better identification. An ADI represents a potential multi-purpose area measure for learning health systems useful in tailoring care.

## Additional File

The additional file for this article can be found as follows:

10.5334/egems.191.s1 Appendix 1 Selected Health Tasks by Health Literacy Score.

**Appendix 1**Selected Health Tasks by Health Literacy Score. DOI: https://doi.org/10.5334/egems.191.s1
