# Supplementary material for: Detecting Risk of Low Health Literacy in Disadvantaged Populations Using Area-based Measures
Source: EGEMS (Wash DC). 2017 Dec 15;5(3):7. doi: 10.5334/egems.191 (PMC5994951; doi:10.5334/egems.191)
Supplement: Appendix 1 — Selected Health Tasks by Health Literacy Score. [file egems-5-3-191-s1.pdf]

Appendix 1. Selected Health Tasks by Health Literacy Score[21]

Health Literacy Score	Percent of Population	Activity
Proficient (310-500)	12%	• Calculating one's personal share of employer health costs using a table<br>• Finding definitions for complex medical terms<br>• Interpreting legal documents and applying to specific health care situations
Intermediate (226-309)	53%	• Determining a health weight range for one's height based on a body mass index<br>• Finding the age range for certain childhood vaccines using a recommendation chart<br>• Determining the times at which one can take a medication based on instructions on the prescription label<br>• Identifying three substances that may interact negatively with an over-the-counter drug to produce side effects, based on the information provided on the package
Basic (185-225)	22%	• Interpreting a clearly-written pamphlet to determine two reasons a person should be testing for a disease even without symptoms<br>• Explaining how a chronic disease may be asymptomatic based on a one-page article about the medical condition
Below Basic (0-184)	14%	• Interpreting a clearly-written pamphlet to determine how often one should have a certain medical test<br>• Identifying what one can and cannot drink before a medical test based on a short set of instructions<br>• Circle the date of a medical appointment on an appointment slip
